# Similar Weight Loss Induces Greater Improvements in Insulin Sensitivity and Liver Function among Individuals with NAFLD Compared to Individuals without NAFLD

**DOI:** 10.3390/nu11030544

**Published:** 2019-03-04

**Authors:** Ruth Schübel, Tobias Nonnenmacher, Disorn Sookthai, Sandra Gonzalez Maldonado, Solomon A. Sowah, Oyunbileg von Stackelberg, Christopher L. Schlett, Mirja Grafetstätter, Diana Nabers, Theron Johnson, Romy Kirsten, Cornelia M. Ulrich, Rudolf Kaaks, Hans-Ulrich Kauczor, Tilman Kühn, Johanna Nattenmüller

**Affiliations:** 1 German Cancer Research Center (DKFZ), Division of Cancer Epidemiology, Im Neuenheimer Feld 581, 69120 Heidelberg, Germany; Ruth.Schuebel@med.uni-heidelberg.de (R.S.); d.sookthai@dkfz-heidelberg.de (D.S.); s.gonzalezmaldonado@dkfz-heidelberg.de (S.G.M.); s.sowah@dkfz-heidelberg.de (S.A.S.); mi.graf@dkfz-heidelberg.de (M.G.); t.johnson@Dkfz-Heidelberg.de (T.J.); r.kaaks@Dkfz-Heidelberg.de (R.K.); 2 Heidelberg University Hospital, Diagnostic and Interventional Radiology, Im Neuenheimer Feld 110, 69120 Heidelberg, Germany; tno144@googlemail.com (T.N.); oyunbileg.stackelberg@med.uni-heidelberg.de (O.v.S.); Christopher.Schlett@med.uni-heidelberg.de (C.L.S.); Hans-Ulrich.Kauczor@med.uni-heidelberg.de (H.-U.K.); 3 German Cancer Research Center (DKFZ), Division of Medical and Biological Informatics, Im Neuenheimer Feld 280, 69120 Heidelberg, Germany; d.nabers@dkfz-heidelberg.de; 4 National Center for Tumor Diseases (NCT), Liquid Biobank, Im Neuenheimer Feld 460, 69120 Heidelberg, Germany; romy.kirsten@nct-heidelberg.de; 5 Huntsman Cancer Institute and Department of Population Health Sciences, University of Utah, 2000 Circle of Hope, Salt Lake City, UT 84112-5550, USA; neli@hci.utah.edu

**Keywords:** non-alcoholic fatty liver disease, magnetic resonance imaging, obesity, liver function, insulin sensitivity

## Abstract

Background: Preliminary evidence suggests that weight loss among obese has differential metabolic effects depending on the presence of non-alcoholic fatty liver disease (NAFLD). We assessed whether NAFLD predisposes to differential changes in liver fat content, liver function, and metabolic parameters upon diet-induced weight loss in a 50-week intervention trial. Methods: 143 overweight and obese non-smokers underwent a 12-week dietary intervention and a 38-week follow-up. Diet-induced changes in anthropometric measures, circulating biomarkers, and magnetic resonance (MR)-derived liver fat content and adipose tissue volumes were evaluated by mixed linear models stratifying by NAFLD at baseline. Results: The prevalence of NAFLD at baseline was 52%. Diet-induced weight loss after 12 (NAFLD: 4.8 ± 0.5%, No NAFLD: 5.1 ± 0.5%) and 50 weeks (NAFLD: 3.5 ± 0.7%, No NAFLD: 3.5 ± 0.9%) was similar in both groups, while the decrease in liver fat was significantly greater in the NAFLD group (week 12: 32.9 ± 9.5% vs. 6.3 ± 4.0%; week 50: 23.3 ± 4.4% vs. 5.0 ± 4.2%). Decreases in biomarkers of liver dysfunction (GGT, ALT, AST) and HOMA IR were also significantly greater in the NAFLD group. Other metabolic parameters showed no significant differences. Conclusion: Our data suggest that individuals with NAFLD show greater improvements of liver function and insulin sensitivity after moderate diet-induced weight loss than individuals without NAFLD.

## 1. Introduction

Enlarged visceral adipose tissue (VAT) depots and ectopic fat deposition contribute to the development of obesity-associated metabolic changes [1]. In particular, fat accumulation in the liver is related to insulin resistance and subclinical inflammation [2,3]. Non-alcoholic fatty liver disease (NAFLD), the most common subtype of liver fat accumulation, develops in individuals without excessive alcohol consumption, use of steatogenic medication or strong genetic predispositions [4]. It is estimated that 30% to 90% of the individuals with obesity (BMI >30 kg/m^2^) also have NAFLD [5]. Given the high prevalence of obesity worldwide and the positive association between obesity and NAFLD, it is expected that the worldwide prevalence of NAFLD will rise well beyond the current level of approximately 20% to 30% reported for the United States and for several regions in Asia [2,6]. NAFLD potentially progresses into non-alcoholic steatohepatitis, and the latter into cirrhosis and hepatocellular carcinoma. Additionally, it has been suggested that NAFLD is a risk factor of cardiovascular diseases and extra-hepatic cancers [7,8].

Th main treatment options to reduce NAFLD associated disease risks are lifestyle interventions implementing changes in dietary habits, together with the goal to induce weight loss and maintain a healthy body weight [9]. Although the liver rapidly responds to short-term caloric restriction (CR) with a reduction in intrahepatic triglyceride storage, even within 48 h [10,11], the sustainability of such effects and mid-term changes in liver fat content, liver function, and metabolic parameters with CR are less well understood. Furthermore, findings indicating that the association between liver fat content and insulin resistance is independent of visceral fat mass [12,13] provide evidence to suggest that the presence or absence of NAFLD in obesity may be an important explanation underlying the phenomena of metabolically healthy or unhealthy obesity [14].

In this project, we analyzed data from a comprehensive dietary weight loss trial including a 12-week intervention phase and a subsequent follow-up with final assessment one year after baseline. We explored whether individuals with NAFLD at baseline would show greater metabolic improvements upon weight loss including changes in liver fat content, liver function and circulating biomarkers of lipid metabolism, glucose metabolism, adipokine signaling, and inflammation than individuals without NAFLD.

## 2. Materials and Methods

### 2.1. Study Design and Study Population

The project presented here is ancillary to the HELENA trial (trial registration number: NCT02449148 ClinicalTrials.gov), a randomized dietary intervention study that has been carried out at the German Cancer Research Center (DKFZ), Heidelberg, and the Heidelberg University Hospital [15]. The study was approved by the ethics committee of the medical faculty Heidelberg (University of Heidelberg, Germany) prior to enrollment. Briefly, 150 overweight and obese (BMI ≥25 kg/m^2^ to <40 kg/m^2^) non-smokers (50% female) in the age range between 35 and 65 years without severe chronic diseases (diabetes, major cardiovascular diseases, cancer, hepatic or kidney dysfunction) were recruited in Heidelberg and surrounding areas between May 2015 and May 2016. Participants were randomly assigned to one of the three dietary groups (intermittent calorie restriction, continuous calorie restriction, or control group) for a 12-week intervention phase, a 12-week maintenance phase, and a 26-week follow-up phase after written informed consent had been obtained. The official guidelines of the German Society for Nutrition for a healthy and balanced diet were provided to all study groups [16,17].

Participants attended examinations at the study center at baseline, after the controlled intervention phase of 12 weeks, and at the follow-up examination 50 weeks after study start. These included anthropometric measurements, blood draw, and magnetic resonance (MR) imaging for body composition analysis and quantification of liver fat content. Body weight, body height, and waist circumference were measured following standard operating procedures by trained personnel after an overnight fast. Seven-day dietary records were completed by all study participants at baseline and week 12. For the MR imaging assessment, typical contraindications were considered (i.e., claustrophobia, cardiac pacemakers or defibrillators, non-removable electronic implants or devices, non 1.5 Tesla-MR imaging approved medical foreign bodies, implants and orthopedic foreign bodies, joint end prostheses, or other metallic foreign bodies) and a separate informed consent for this module was obtained from 145 participants of the HELENA trial. Details on the study flow, drop-out rates, and available dataset used for the presented analysis stratified by NAFLD at study start (MR-derived liver fat ≤5.56% vs. liver fat >5.56%) are shown in Figure S1. Overall, 143 participants were included in the present analyses. 

For the present post hoc analyses on metabolic responses to weight loss depending on the presence of NAFLD, we used data of the HELENA trial (trial registration number: NCT02449148 ClinicalTrials.gov), a dietary intervention trial initially carried out to investigate the metabolic effects of intermittent vs. continuous calorie restriction. The main results of the HELENA trial with respect to the pre-specified primary and secondary outcomes have previously been published [18], and the methodological aspects of the trial have been described in detail in two previous publications [15,18].

### 2.2. Laboratory Analyses

All blood collection was obtained after an overnight fast of at least 8 h. Clinical routine markers (alanine transaminase (ALT), aspartate transaminase (AST), γ-glutamyl transpeptidase (GGT), high density lipoprotein (HDL), low density lipoprotein (LDL), fasting glucose, HbA1c) were measured by routine assays directly after blood draw at the Central Laboratory of the Heidelberg University Hospital which is a certified clinical chemistry laboratory. Remaining samples were processed and frozen at −80 °C. Plasma levels of insulin, leptin, resistin, and C-reactive protein (CRP) were measured by electrochemiluminescence immunoassays (ECLIA) on the “Quickplex SQ 120” instrument from Meso Scale Discoveries (MSD, MD, Rockville, Maryland, USA), using singleplex kits from MSD in the laboratory of the Division Cancer Epidemiology at German Cancer Research Center (DKFZ), Heidelberg. At baseline and after week 12 differences in the expression of 82 preselected genes gained from the subcutaneous adipose tissue (SAT) (see Supplemental Table S2 in a previous publication [18]) were assessed. 

Details on the collection and processing of all laboratory analyses and biospecimens (blood, SAT) are described previously [18]. 

### 2.3. MR Imaging

MR imaging was performed at a 1.5 Tesla MR scanner (MAGNETOM Aera; Siemens Healthcare, Erlangen, Germany) to assess abdominal VAT and subcutaneous adipose tissue (SAT) volume as well as liver fat content. Hardware applied MR-protocol and software remained constant between all MR-scans for this study. 

Liver fat content was quantified using a multi-echo GRE technique (Siemens LiverLab, Siemens Healthcare, Erlangen, Germany) [15,19,20,21,22]. Within the proton density fat fraction map, three regions of interest (each 4 cm^2^) were placed dorsally, anterior-medially, and anterior-laterally of the right liver lobe at a level immediate cranial of the liver hilum, while larger vessels and connective tissue were avoided using dedicated software (OsiriX, Pixmeo SARL, Bernex, Switzerland) [19]. The positioning of the three regions of interest was kept constant for the three repeated measurements (baseline, week 12 and week 50) of each study participant. An intra-/inter-reader reproducibility analysis including 32 cases showed a high reproducibility with an ICC of 0.99 and 0.99, respectively.

A 2-point DIXON sequence covering from the neck to the upper legs was applied for SAT and VAT volume quantification. The quantification was performed semi-automatically using an in-house developed algorithm based on the Medical Imaging Interaction Toolkit (MITK), as described previously [23].

### 2.4. Statistical Analyses

The main objective of the HELENA trial was to investigate whether intermittent calorie restriction has greater effects on adipose tissue gene expression, body composition, and metabolic parameters than continuous calorie restriction. Statistical analyses did not indicate differences between intermittent and continuous calorie restriction with respect to the pre-specified metabolic, anthropometric and body composition outcomes [18]. For the present project, we analyzed whether the overall intervention effects (i.e., changes in liver fat, liver function, and metabolic parameters induced by any of the three dietary regimens), differed between individuals with NAFLD (>5.56% MR-derived liver fat content) vs. individuals without NAFLD (≤5.56% MR-derived liver fat content) at baseline [4]. Using a cut-off of 5.56% was recommended by Szczepaniak et al. in a patient cohort of the Dallas Heart Study and evaluated by Kramer et al. also for proton-density fat-fraction MRI [4,24]. A cross-classification of the initial study arm and NAFLD prevalence indicating an equal distribution of NAFLD across the groups is presented in Supplementary Figure S2. Our study results were highly similar using an alternative cut-point for NAFLD at 5%, which has also been suggested by Brunt et al. and Hetterich et al. using the proton-density fat-fraction MRI [5,25]. In fact, there were only two individuals with liver fat values between 5% and 5.56%, which were re-classified in this sensitivity analysis (data not shown). 

Linear mixed models for repeated measurement, with age and sex adjustment, were carried out to analyze time by group (NAFLD, no NAFLD) interaction effects for baseline to week 12, and baseline to week 50. The magnitudes of change in outcome measures i.e., liver fat, liver function (AST, ALT, GGT), and metabolic parameters (homeostatic model of insulin resistance (HOMA-IR), insulin, glucose, HbA1c, LDL, HDL, leptin, resistin, CRP) over time are shown as relative differences with baseline values as the reference.

Ancillary cross-sectional correlations between liver fat content, BMI, waist circumference, VAT volume, and SAT volume were analyzed by Pearson’s correlation coefficients with and without age and sex adjustment. Both, VAT and SAT volumes were height standardized with the residual method [26] prior to analyses. SAS 9.4 (Cary, NC, USA) was used for all statistical analyses.

Pre-processing of microarray data (log2 transformation, imputation of missings [27], batch standardization by ComBat [28]) was conducted in Chipster 3.8. The analyses on gene expression profiles per NAFLD categories were obtained on the basis of linear models with age and sex adjustment, using the limma package in R (R Foundation for Statistical Computing) including Benjamini Hochberg correction for multiple testing.

## 3. Results

### 3.1. Characteristics of the Study Population

**Figure 1:**
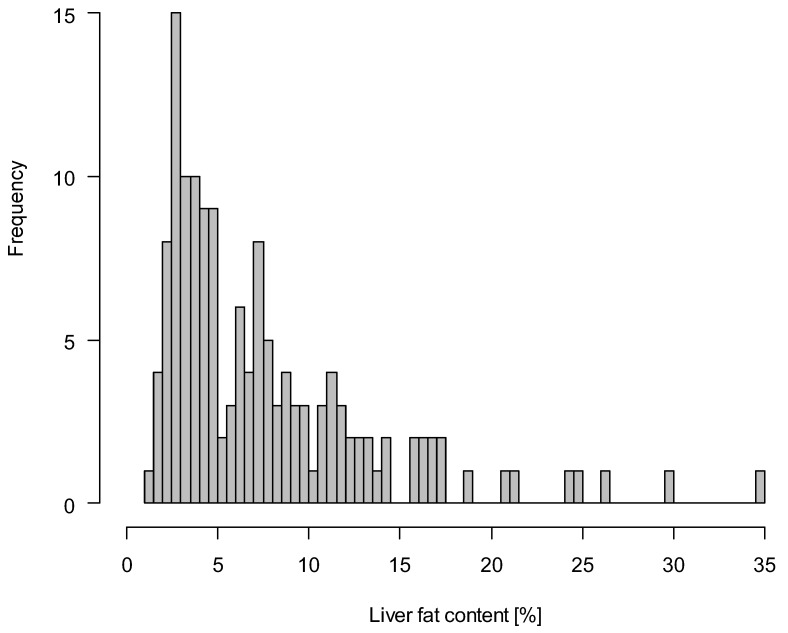
Histogram of MR-derived liver fat content (%) at baseline (*n* = 143). MR: magnetic resonance.

Among the study cohort (*n* = 143) of overweight and obese individuals, 52.4% (*n* = 75) had NAFLD (>5.56% MR-derived liver fat content) at baseline. The majority of the individuals in the NAFLD group had liver fat contents below 15%, and one person had a liver fat content >30% (Figure 1). Mean age was 50.0 ± 8.1 years among participants with NAFLD, and 50.1 ± 8.1 years among participants without NAFLD (Table 1). The prevalence of NAFLD was higher among men (61.1%) than women (43.6%). Average BMI, VAT volume, SAT volume, and circulating biomarker levels were higher in the NAFLD group compared to the group without NAFLD at baseline (Table 1). 

### 3.2. Correlations pf Liver Fat with Anthropometric Measures and Body Fat Volumes

**Figure 2:**
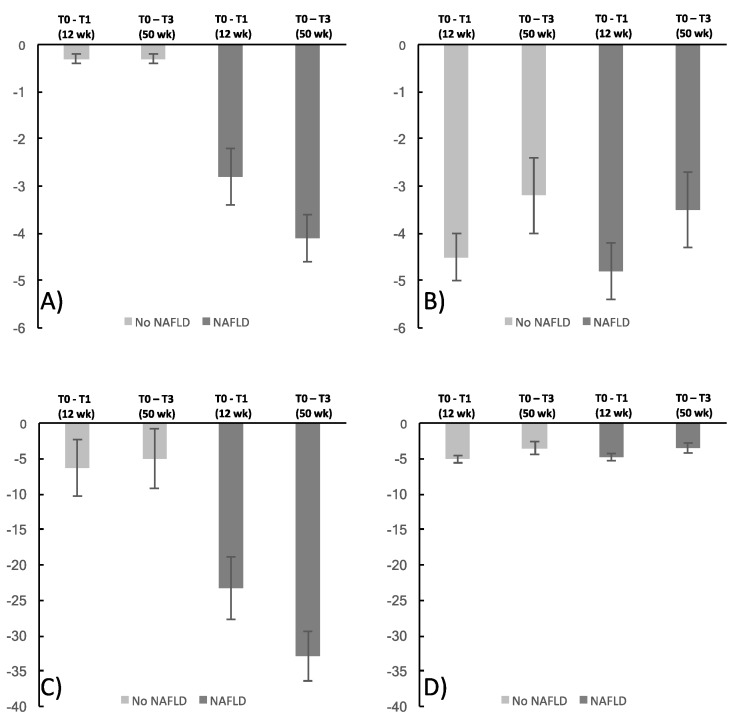
Mean absolute and mean relative changes (standard errors) in liver fat content and body weight over 12 weeks and 50 weeks among individuals with and without NAFLD. (**A**) Absolute changes in liver fat content (%), (**B**) Absolute changes in body weight (kg), (**C**) Relative changes in liver fat content (%), (**D**) Relative changes in body weight (%).

Values of absolute change of body weight and liver fat content are given in Figure 2. Correlations of liver fat content with BMI, waist circumference, height standardized VAT volume, and height standardized SAT volume at baseline are visualized in Figure S4. Pearson´s correlation coefficients were highest for liver fat and waist circumference (*p* < 0.01; *r* = 0.45), followed by liver fat and VAT volume (*p* < 0.01; *r* = 0.39), liver fat and BMI (*p* < 0.01; *r* = 0.27), and liver fat and SAT (*p* = 0.01; *r* = 0.20). Adjustment for age and sex did not substantially change the results (*r* = 0.40, ρ = 0.38, *r* = 0.29, and *r* = 0.30, respectively). Cross-classification of BMI categories (overweight: BMI ≥25 kg/m^2^ to ≤30 kg/m^2^ and obese: BMI >30 kg/m^2^ to <40 kg/m^2^) and NAFLD prevalence indicated that 26.7% of the individuals with NAFLD and 52.9% without NAFLD were overweight, whereas 73.3% and 47.1%, respectively were obese at baseline (see Figure S3).

### 3.3. NAFLD and Metabolic Effects of Weight Loss

**Figure 3:**
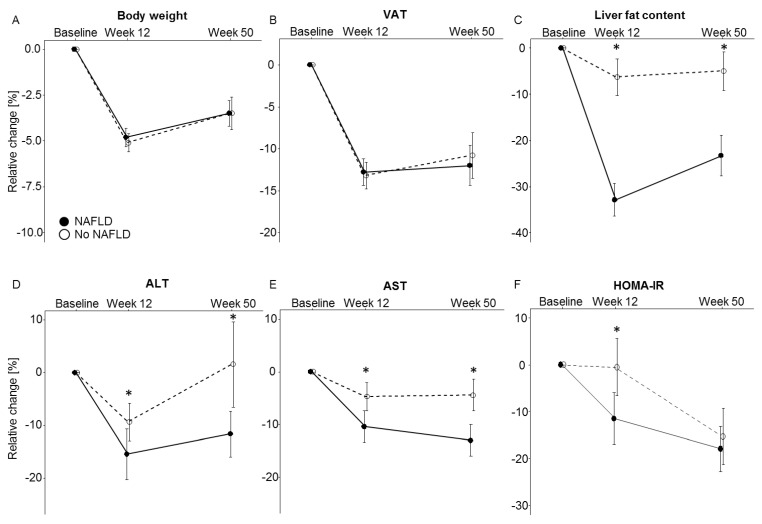
Relative change in (**A**): body weight, (**B**): visceral adipose tissue (VAT), (**C**): liver fat content, (**D**): alanine transaminase (ALT), (**E**): aspartate transaminase (AST), and (**F**): HOMA-IR for individuals with NAFLD and without NAFLD at baseline. Data are shown as mean ± SE of relative differences over time with baseline values as the reference. Significant (*p* < 0.05) time by group interactions, again with baseline values as the reference are depicted with a star (*).

Relative changes in anthropometric measures, body fat volumes, metabolic biomarker levels, and dietary composition between baseline and week 12 and baseline and week 50 are shown in Table 2 and Figure 3. Absolute values for baseline, week 12, and week 50 are summarized in Table S1. Weight changes between baseline and week 12 (NAFLD: 5.1 ± 0.5%; no NAFLD: 4.8 ± 0.5%), and between baseline and week 50 (NAFLD: 3.5 ± 0.9%; no NAFLD: 3.5 ± 0.7%) were similar in both groups (week 12: *p* = 0.80; week 50: *p* = 0.84). The observed weight loss across the intervention phase and slight trend for weight regain across the follow-up phase was paralleled by proportional changes in VAT and SAT volumes, again without significant differences between the study groups (*p* > 0.05). In contrast, the relative decrease in liver fat content across the dietary intervention phase (32.9 ± 9.5%) and follow-up phase (23.3 ± 4.4%) was significantly greater (*p* < 0.01) in the NAFLD group as compared to the group without NAFLD at baseline (6.3 ± 4.0% and 5.0 ± 4.2% respectively). 

Relative changes in liver function tests including AST, ALT, and GGT were significantly greater in the NAFLD group at week 12 and week 50 (all *p* < 0.05). Furthermore, HOMA-IR values decreased significantly greater in the NAFLD group (11.5 ± 5.5%) than in the group without NAFLD (0.5 ± 6.1%; *p* = 0.04) at week 12, while a similar, but non-significant trend for a difference in insulin levels was observed (NAFLD: 7.9 ± 5.2%; no NAFLD: 2.6 ± 5.9%, *p* = 0.06). As opposed to the differences in liver function markers and liver fat, the differences in HOMA-IR and insulin levels were attenuated and statistically non-significant at week 50. Other metabolic parameters (LDL, HDL, fasting glucose, HbA1c, leptin, resistin, CRP) showed no significant differences between the groups at any time point. None of the findings on differential changes in biomarkers between individuals with vs. without NAFLD were affected by statistical adjustment for BMI or VAT values. 

Overall, there were no significant differences for change in gene expression levels between baseline and week 12.

## 4. Discussion

In the present study, we assessed if overweight or obese individuals with NAFLD at baseline experienced greater metabolic improvements with dietary weight loss than overweight or obese individuals without NAFLD. Despite highly similar mean weight loss in both groups, individuals with NAFLD showed significantly greater decreases in liver fat content and liver function markers over the 12-week intervention phase. These differences were still observed after the follow-up phase, i.e., 50 weeks after baseline. Decreases in HOMA-IR consistent with improvement in insulin-sensitivity were also greater among individuals with NAFLD at week 12, although this difference was attenuated and did not remain statistically significant throughout follow-up. No differences in intervention effects depending on NAFLD at baseline were observed regarding parameters of glucose metabolism (fasting glucose, HbA1c), lipid metabolism (HDL, LDL), adipokine signaling (leptin, resistin) or inflammation (CRP).

We observed moderate correlations of liver fat with BMI, waist circumference, and VAT volume. Cross-classification of NAFLD with BMI categories indicated a tendency for a higher NAFLD prevalence among obese than overweight participants. However, NAFLD prevalence was also high in non-obese study participants. As growing evidence suggests that NAFLD is more closely linked to insulin resistance than visceral fat mass [12,13,29], these observations are in line with the notion that NAFLD may help to discriminate metabolically healthy vs. unhealthy overweight and obesity beyond existing anthropometric and imaging parameters of total and abdominal fat accumulation [30].

The present analyses indicated that NAFLD is a determinant of the success of dietary weight loss interventions with regard to improvements in liver function and insulin sensitivity. Although mean relative changes in body weight, VAT volume, and SAT volume were similar between individuals with and without NAFLD in the present analyses, the decrease in liver fat content was more than four times higher in the NAFLD group. Similarly, there were significantly greater decreases in liver function parameters (AST, ALT, and GGT) and improvements of insulin sensitivity (HOMA-IR) among individuals with NAFLD compared to individuals without NAFLD at baseline. The finding on greater changes in liver fat and liver function parameters among individuals with NAFLD are comparable to the results of two previous studies, each with a duration of six months, the B-SMART study (*n* = 50), a randomized trial initially designed to investigate differences between a low-carbohydrate and low-fat diet for weight loss [31], and a multimodal weight loss intervention trial among severe obese individuals (*n* = 129) from the RENEW trial [32]. Our finding on a significantly greater decrease in HOMA-IR levels in the NAFLD group was not observed in either of the prior trials, although, severely obese individuals (mean BMI > 42) with NAFLD at baseline showed significantly greater decreases in fasting glucose levels with weight loss than obese individuals without NAFLD in the RENEW trial [32]. By contrast, there was no differential change in glucose levels in our study. In addition, Tiikkainen et al. showed in a study among 23 women with gestational diabetes greater reductions in liver fat among women with initially higher liver fat values (i.e., NAFLD) at similar weight loss, which is in line with our results but in a smaller study group of only woman with gestational diabetes [33]. 

With regard to HOMA-IR, we have to acknowledge that the differential changes depending on the presence of NAFLD after the intervention phase in the present analyses were no longer observed after follow-up. Nevertheless, it should be noted that our study cohort only included overweight and obese individuals with glucose levels within the reference ranges and without a diagnosis of Diabetes type 2 at baseline. Thus, it is likely that the present differences in HOMA-IR may be greater among individuals with evident metabolic impairments at baseline. There is a lack of an established guideline for non-invasive diagnosis of NAFLD, whereas liver fat contents >5.56% [4] or >5% [5] are frequently used thresholds. Yet, our results remained highly similar, irrespective of the selected cut-point for classification of NAFLD. We acknowledged that the evaluation of liver fat content relied on MR-derived estimates as it was not possible to perform biopsies of the liver parenchyma because of the risk of complications, also we did not evaluate the sensitivity of our liver fat assessment directly against MRS or biopsy. However, liver fat quantification using a multi-echo GRE technique with the proton density fat fraction is a biopsy proven, highly reliable and accurate noninvasive method [34,35]. Yokoo et al. described a sensitivity of 0.950 with a specificity of 1.000 for the multi-echo Dixon technique, which they assessed also for several threshold values between 5%–8% fat content [22]. Tang et al. stated a sensitivity of 97% with 100% specificity to distinguish patients with steatosis grade 0 (<5%) from patients with steatosis grade 1 or greater (>5%) [21]. We did not have information on genetic risk factors playing a role in progression and severity of NAFLD, which may be considered in future personalized treatment approaches [36], although the variation in liver fat explained by known genetic mutations is rather low [30]. As this study is a post hoc analysis of NAFLD vs. no-NAFLD, the grouping and randomization of the original trial were abandoned. After 12 weeks of intervention weight loss (defined as loss of weight >5%) showed following distribution in the three original study groups: intermittent calorie restriction: *n* = 29 (44.6%), continuous calorie restriction: *n* = 23 (35.4%) and control group: *n* = 13 (20.0%), for further details see in our recent publication [18]. However, this has no effect on the present study.

In summary, participants with NAFLD at baseline showed greater changes in liver fat content and improvements in liver function and insulin sensitivity with moderate diet-induced weight loss than individuals without NAFLD. This finding suggests that overweight or obese individuals with NAFLD benefit more strongly from weight loss interventions than individuals without NAFLD. Thus, our results underline that weight loss induced by dietary restriction should be considered as the first line of intervention in the prevention of NAFLD progression and its possible complications, especially in the absence of routine medical treatment [37].

## Figures and Tables

**Table 1:** Characteristics of the study cohort at baseline.

	No NAFLD at Baseline	NAFLD at Baseline
*n* (women/men)	68 (40/28)	75 (31/44)
Age, years	50.7 (44.1,56.8)	49.7 (44.0,57.3)
Weight, kg	89.4 (79.7,95.7)	99.4 (87.7,110.0)
BMI, kg/m^2^	29.4 (27.5,33.0)	32.7 (29.5,34.6)
Waist circumference, cm	99.8 (90.8,105.8)	110.0 (101.5,115.0)
Fat distribution		
VAT, L	3.6 (2.6,4.9)	5.8 (4.4,7.1)
SAT, L	10.6 (8.9,14.0)	12.9 (10.0,14.8)
Liver fat content, %	3.4 (2.6,4.2)	9.8 (7.2,13.5)
Liver tests		
ALT, μkat/L	0.32 (0.27,0.4)	0.5 (0.38,0.6)
AST, μkat/L	0.35 (0.3,0.41)	0.38 (0.35,0.47)
GGT, μkat/L	0.32 (0.24,0.47)	0.43 (0.3,0.67)
Metabolism		
Glucose, mmol/L	5.11 (4.8,5.3)	5.3 (5.0,5.6)
Insulin, pmol/L	60.4 (47.2,84.0)	88.2 (64.6,145.8)
HOMA-IR	1.9 (1.5,3.0)	3.1 (2.2,4.8)
HbA1c, %	5.4 (5.2,5.6)	5.6 (5.3,5.7)
LDL, mmol/L	3.2. (2.6,3.7)	3.3 (2.9,3.7)
HDL, mmol/L	1.4 (1.2,1.7)	1.2 (1.0,1.4)
Adipokines		
Leptin, μg/L	18.7 (8.6,35.6)	16.0 (8.6,29.4)
Resistin, μg/L	5.4 (4.3,6.5)	5.2 (3.8,6.5)
CRP, nmol/L	24.8 (12.4,45.7)	28.6 (15.2,54.3)
Alcohol intake, g/day ^a^	2.2 (0.3,4.3)	1.9 (0.3,5.8)

Data are shown as median (25 percentile, 75 percentile). ^a^ Self-reported alcohol intake is calculated from 7-day dietary records completed at baseline. Abbreviations: ALT, alanine transaminase; AST, aspartate transaminase; GGT, gamma-glutamyl transpeptidase; HDL, high density lipoprotein; HOMA-IR, homeostatic model of insulin resistance; LDL, low density lipoprotein; SAT, subcutaneous adipose tissue; VAT, visceral adipose tissue.

**Table 2:** Relative change (%) in body composition and metabolic biomarkers.

	No NAFLD at Baseline (*n* = 68)		NAFLD at Baseline (*n* = 75)		*p* value ^a^	
	Baseline to week 12	Baseline to week 50	Baseline to week 12	Baseline to week 50	Baseline to week 12	Baseline to week 50
Weight	−5.1 ± 0.5	−3.5 ± 0.9	−4.8 ± 0.5	−3.5 ± 0.7	0.80	0.84
Waist circumference, cm	−4.8 ± 0.6	−1.7 ± 0.8	−4.3 ± 0.6	−2.3 ± 0.7	0.85	0.59
Fat distribution						
VAT, L	−13.2 ± 2	−10.8 ± 2.7	−12.8 ± 1.6	−12.0 ± 2.4	0.08	0.23
SAT, L	−9.8 ± 1.4	−5.8 ± 2.2	−10.6 ± 1.3	−7.5 ± 1.9	0.36	0.65
Liver fat content, %	−6.3 ± 4.0	−5.0 ± 4.2	−32.9 ± 9.5	−23.3 ± 4.4	<0.01	<0.01
Liver function						
ALT, μkat/L	−9.3 ± 3.6	1.6 ± 8.8	−15.4 ± 4.8	−11.6 ± 4.3	<0.01	0.02
AST, μkat/L	−4-7 ± 2.7	−4.4 ± 3.0	−10.4 ± 3.0	−13.0 ± 3.0	0.03	0.02
GGT, μkat/L	−17.2 ± 2.5	−0.2 ± 7.9	−20.8 ± 3.6	−14.7 ± 3.3	0.01	0.03
Metabolism						
Glucose, mmol/L	−4.3 ± 1.2	−5.3 ± 1.1	−5.5 ± 0.9	−4.4 ± 1.0	0.45	0.44
Insulin, pmol/L	2.6 ± 5.9	−12.2 ± 5.6	−7.9 ± 5.2	−15.8 ± 4.7	0.06	0.08
HOMA-IR	−0.5 ± 6.1	−15.3 ± 6.0	−11.5 ± 5.5	−18.0 ± 4.9	0.04	0.08
HbA1c, %	0 ± 0.5	−1.1 ± 0.5	−0.7 ± 0.4	−2.3 ± 0.5	0.25	0.08
LDL, mmol/L	−9.1 ± 1.6	2.6 ± 2.3	−4.5 ± 2.0	−0.6 ± 2.3	0.20	0.25
HDL, mmol/L	−9.4 ± 1.6	−2.7 ± 1.7	−8.3 ± 1.7	−0.1 ± 1.7	0.35	0.29
Adipokines						
Leptin, μg/L	−28.8 ± 4.7	−15.2 ± 7.4	−25.9 ± 6.7	−10.7 ± 6.9	0.74	0.89
Resistin, μg/L	−27.3 ± 9.5	4.5 ± 9.0	−28.6 ± 7.0	6.0 ± 5.7	0.59	0.20
CRP, nmol/L	−0.6 ± 9.8	26.9 ± 21.6	12.2 ± 16.4	18.5 ± 19.9	0.58	0.52
Dietary intake ^b^						
Energy intake, kcal	−20.4 ± 2.8	NA	−20.9 ± 2.6	NA	0.61	
Fat, %	−13.5 ± 2.7		−11.9 ± 2.5		0.88	
Carbohydrates, %	9.9 ± 2.5		7.2 ± 2.2		0.46	
Protein, %	15.5 ± 3.3		20.2 ± 3.4		0.34	
Fibers, g/day	24.8 ± 5.8		23.4 ± 5.7		0.63	

Data are shown as mean ± SE of relative differences with baseline values as the reverence. ^a^ *p*-values for time by group interaction effects were calculated using linear mixed models for baseline to week 12 and baseline to week 50. ^b^ Dietary intake data were self-reported in the 7-day dietary record. Abbreviations: ALT, alanine transaminase; AST, aspartate transaminase; GGT, gamma-glutamyl transpeptidase; HDL, high-density lipoprotein; HOMA-IR, homeostatic model of insulin resistance; LDL, low-density lipoprotein; SAT, subcutaneous adipose tissue; VAT, visceral adipose tissue.

## Supplementary Materials

The following are available online at https://www.mdpi.com/2072-6643/11/3/544/s1, Figure S1: Flow chart on the HELENA trial with an indication of data sets used for the stratified analysis by NAFLD (yes/no) at study start. Abbreviations: CCR, continuous calorie restriction; ICR, intermittent calorie restriction; NAFLD non-alcoholic fatty liver disease; SAT, subcutaneous adipose tissue; VAT, visceral adipose tissue, Figure S2: Cross-classification of the study group and prevalence of NAFLD at baseline (*n* = 143). Abbreviations: CCR, continuous calorie restriction and ICR, intermittent calorie restriction, Figure S3: Cross-classification of BMI category (overweight: BMI >25 kg/m^2^ to ≤30 kg/m^2^; obese: BMI >30 kg/m^2^ to <40 kg/m^2^) and prevalence of NAFLD at baseline (*n* = 143), Figure S4: Cross-sectional association of liver fat content and (a): Body mass index, (b): waist circumference, (c): height standardized visceral adipose tissue (VAT), and (d): height standardized subcutaneous adipose tissue (SAT) at baseline. Table S1: Mean values for body composition measures and metabolic biomarker levels at baseline, week 12 and week 50, stratified for the presence of NAFLD at baseline.
