# Supplementary material for: Similar Weight Loss Induces Greater Improvements in Insulin Sensitivity and Liver Function among Individuals with NAFLD Compared to Individuals without NAFLD
Source: Nutrients. 2019 Mar 4;11(3):544. doi: 10.3390/nu11030544 (PMC6471668; doi:10.3390/nu11030544)
Supplement: Supplementary file 1 [file nutrients-11-00544-s001.pdf]

Similar Weight Loss Induces Stronger Improvements in Insulin Sensitivity and Liver Function among Individuals with NAFLD Compared to Individuals without NAFLD

Ruth Schübel[1,2], Tobias Nonnenmacher[2], Disorn Sookthai[1], Sandra Gonzalez Maldonado[1], Solomon A. Sowah[1], Oyunbileg von Stackelberg[2], Christopher L. Schlett[2], Mirja E. Grafestätter[1], Diana Nabers[3], Theron Johnson[1], Romy Kirsten[4], Cornelia M. Ulrich[5], Rudolf Kaaks[1], Hans-Ulrich Kauczor[2], Tilman Kühn[1*], and Johanna Nattenmüller[2*]

[1] German Cancer Research Center (DKFZ), Division of Cancer Epidemiology, Im Neuenheimer Feld 581, 69120 Heidelberg, Germany
[2] Heidelberg University Hospital, Diagnostic and Interventional Radiology, Im Neuenheimer Feld 110, 69120 Heidelberg, Germany
[3] German Cancer Research Center (DKFZ), Division of Medical and Biological Informatics, Im Neuenheimer Feld 280, 69120 Heidelberg, Germany
[4] National Center for Tumor Diseases (NCT), Liquid Biobank, Im Neuenheimer Feld 460, 69120 Heidelberg, Germany
[5] Huntsman Cancer Institute and Department of Population Health Sciences, University of Utah, 2000 Circle of Hope, Salt Lake City, Utah 84112-5550, USA

* Correspondence: Johanna.nattenmueller@med.uni-heidelberg.de; Phone: +49 6221 5636462; t.kuehn@dkfz-heidelberg.de; Phone: +49 6221 423184.

Figures

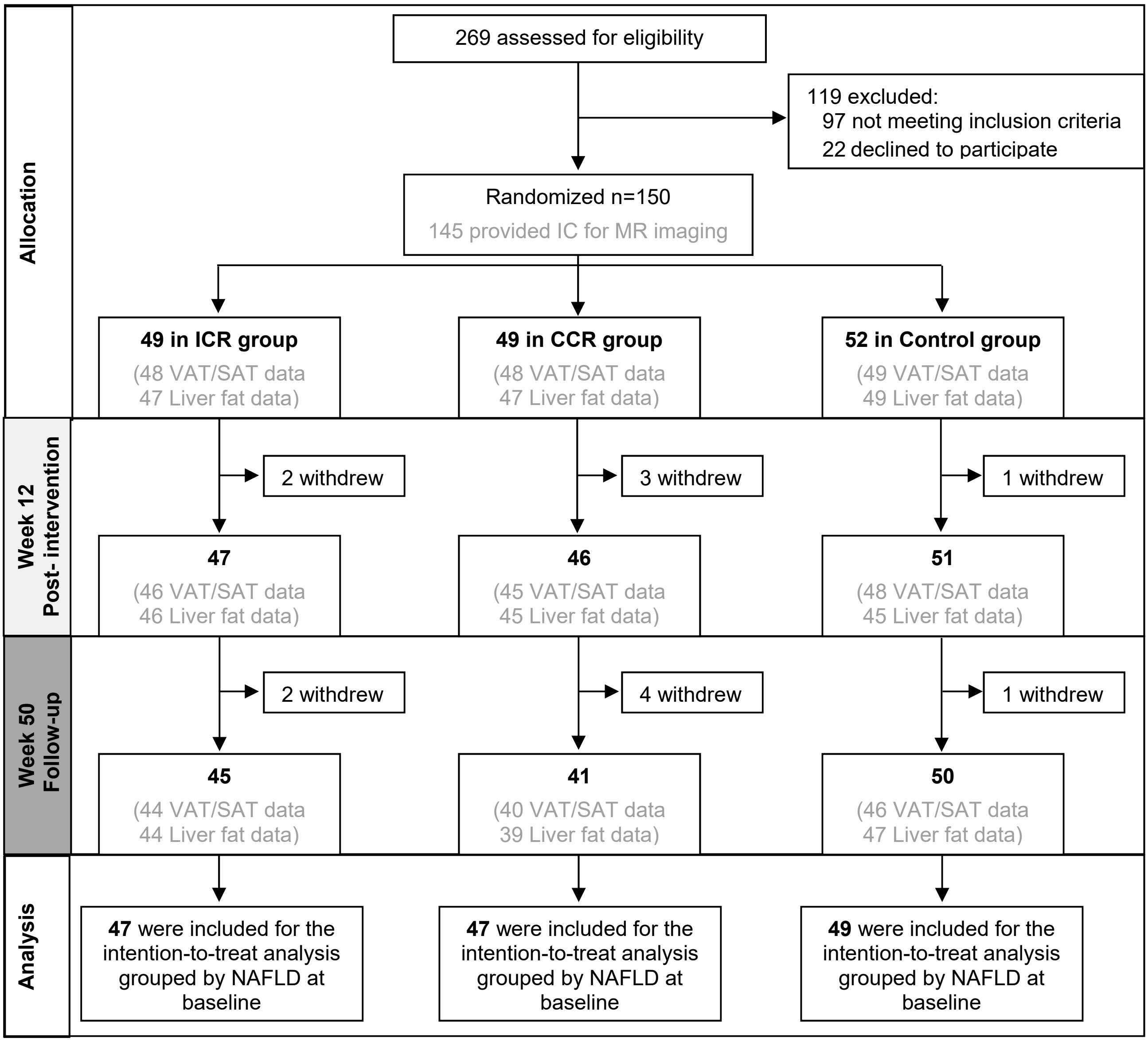

Figure S1: Flow chart on the HELENA Trial with indication of data sets used for the stratified analysis by NAFLD (yes/no) at study start.
Abbreviations: CCR, continuous calorie restriction; ICR, intermittent calorie restriction; NAFLD non-alcoholic fatty liver disease; SAT, subcutaneous adipose tissue; VAT, visceral adipose tissue;

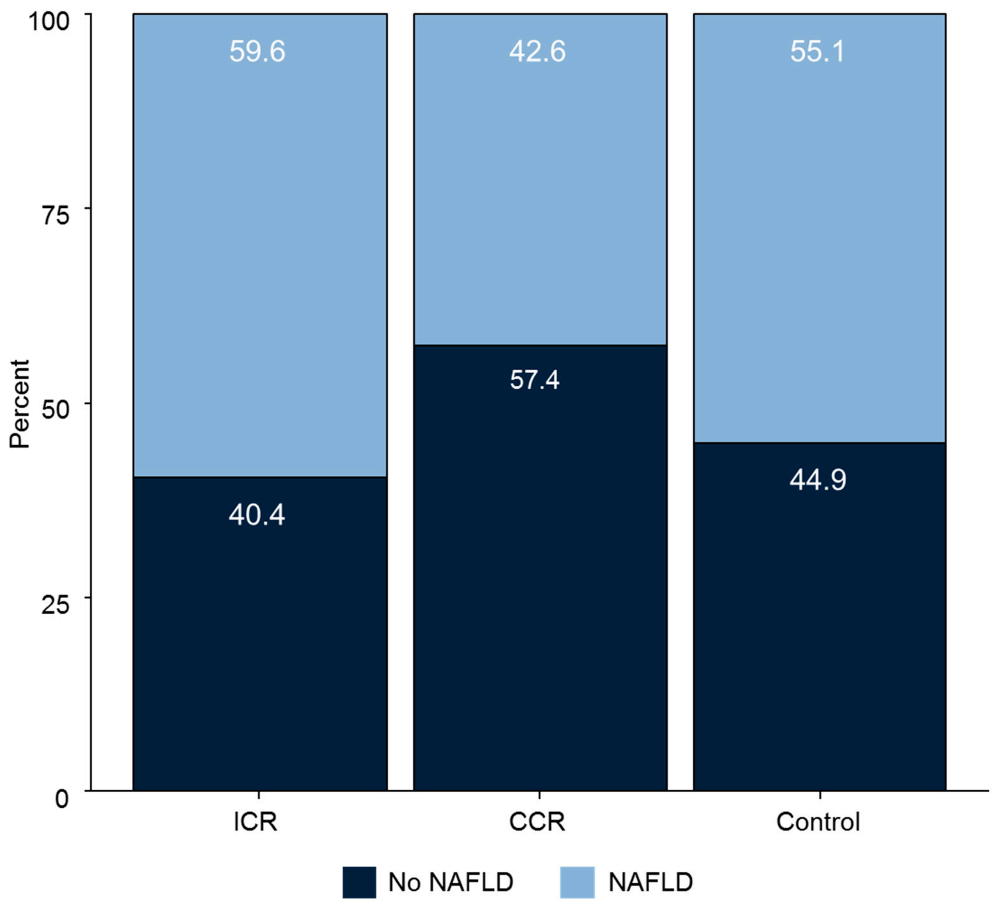

Figure S2: Cross-classification of study group and prevalence of NAFLD at baseline (n=143). Abbreviations: CCR, continuous calorie restriction and ICR, intermittent calorie restriction

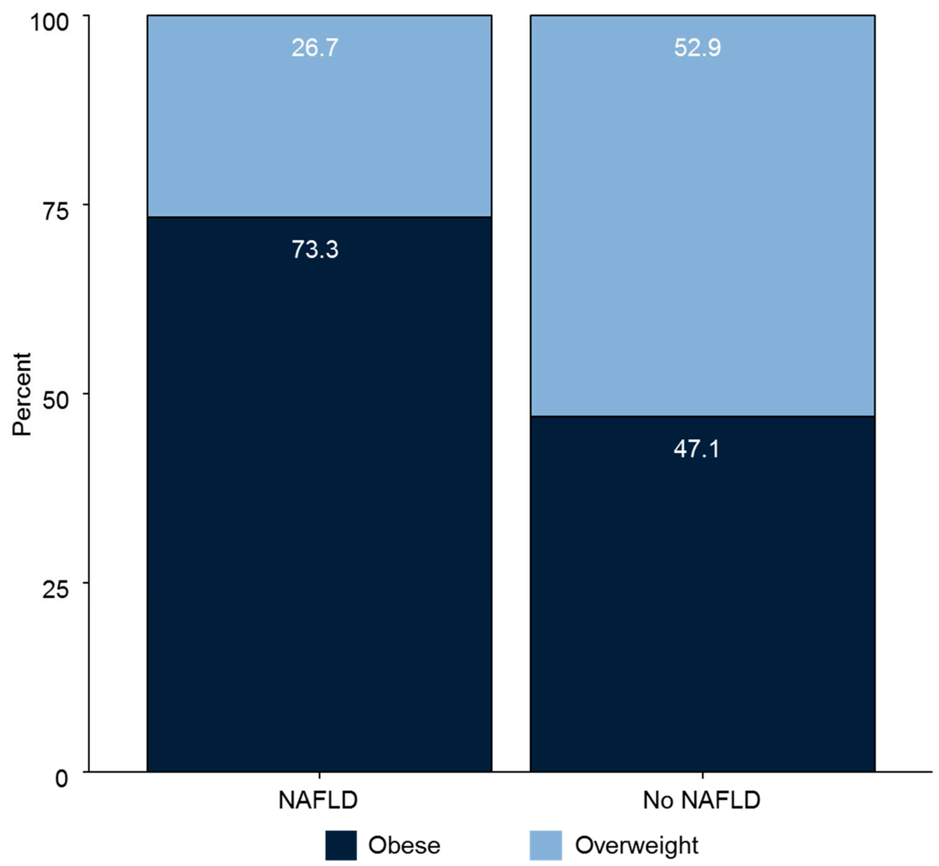

Figure S3: Cross-classification of BMI category (overweight: BMI >25kg/m² to ≤30kg/m²; obese: BMI >30kg/m² to <40kg/m²) and prevalence of NAFLD at baseline (n=143)

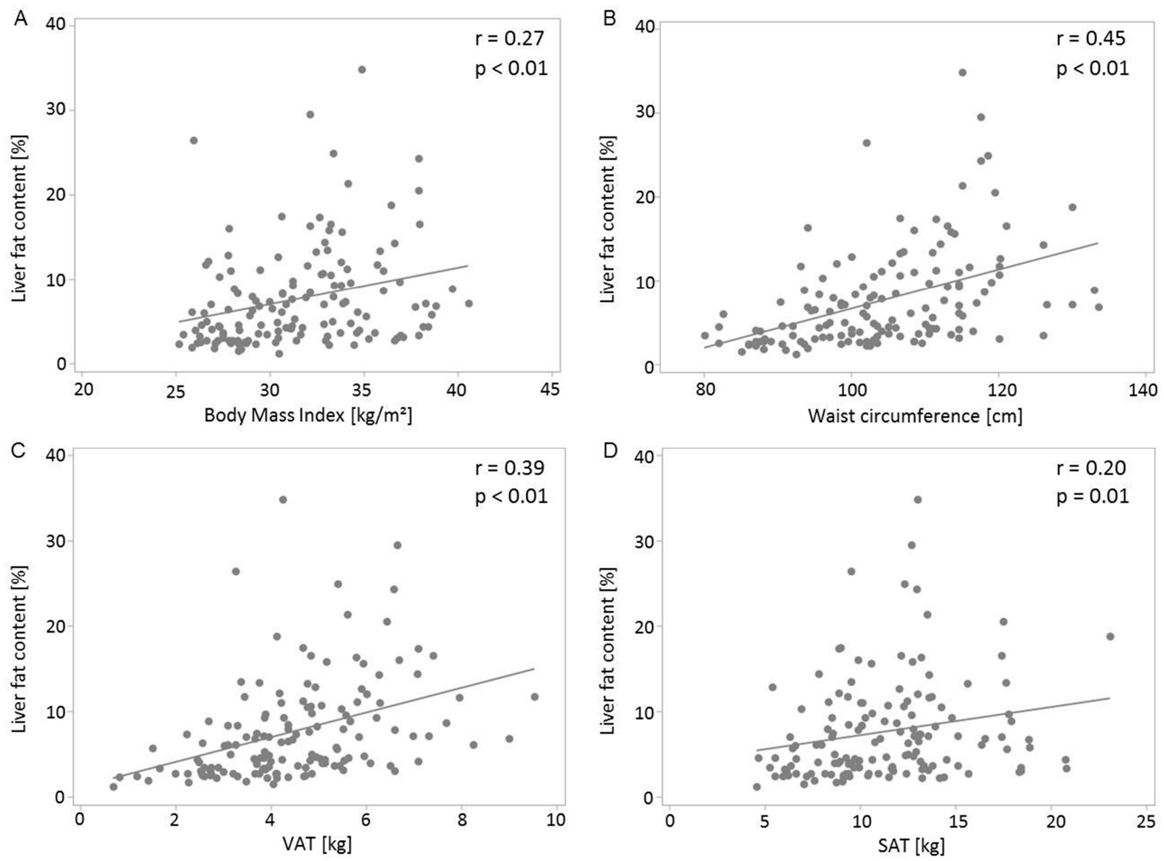

Figure S 4: Cross-sectional association of liver fat content and (**a)**: Body Mass Index, (**b)**: waist circumference, (**c)**: height standardized visceral adipose tissue (VAT), and (**d)**: height standardized subcutaneous adipose tissue (SAT) at baseline.

Tables

Table S1: Mean values for body composition measures and metabolic biomarker levels at baseline, week 12 and week 50, stratified for the presence of NAFLD at baseline

	No NAFLD at baseline (n=68)			NAFLD at baseline (n=75)		
	Baseline	Week 12	Week 50	Baseline	Week 12	Week 50
Weight	88.3 ± 12.7	83.3 ± 11.4	84.6 ± 12.1	99.6 ± 14.9	95.2 ± 14.9	96.4 ± 15.9
BMI, kg/m²	30.3 ± 3.6	28.6 ± 3.4	29.0 ± 3.6	32.4 ± 3.6	31.0 ± 3.9	31.4 ± 4.1
Waist circumference, cm	99.2 ± 10.0	94.2 ± 10.3	97.2 ± 11.2	108.9 ± 10.6	104.5 ± 10.9	107.1 ± 11.5
Fat distribution						
VAT, kg	3.6 ± 1.6	3.1 ± 1.5	3.2 ± 1.6	5.3 ± 1.8	4.6 ± 1.8	4.7 ± 2.1
SAT, kg	10.6 ± 3.8	9.5 ± 3.5	9.6 ± 3.4	12.0 ± 3.6	10.8 ± 3.7	11.1 ± 3.9
Liver fat content, %	3.4 ± 1.0	3.0 ± 1.1	3.1 ± 1.1	11.6 ± 5.9	7.7 ± 4.9	8.9 ± 6.2
Liver tests						
ALT, units/L	21.8 ± 9.2	20.0 ± 9.0	21.3 ± 12.2	31.2 ± 10.8	24.6 ± 10.2	26.9 ± 14.0
AST, units/L	21.3 ± 4.6	20.1 ± 4.5	20.2 ± 5.4	24.5 ± 5.4	21.1 ± 5.1	20.9 ± 7.0
GGT, units/L	22.5 ± 12.5	18.5 ± 10.8	23.1 ± 18.8	30.7 ± 17.5	23.4 ± 14.1	26.7 ± 17.6
Metabolism						
Glucose, mg/dl	91.6 ± 6.8	87.1 ± 8.1	85.8 ± 7.2	95.2 ± 7.9	90.0 ± 8.5	90.9 ± 8.8
Insulin, µU/ml	9.7 ± 4.3	9.0 ± 4.5	7.9 ± 5.4	14.8 ± 7.3	12.5 ± 7.1	11.4 ± 6.7
HOMA-IR	2.2 ± 1.0	2.0 ± 1.1	1.7 ± 1.2	3.5 ± 1.8	2.8 ± 1.7	2.6 ± 1.6
HbA1c, %	5.4 ± 0.3	5.4 ± 0.3	5.3 ± 0.3	5.5 ± 0.3	5.5 ± 0.3	5.4 ± 0.4
LDL, mg/dl	124.1 ± 28.4	113.2 ± 25.6	128.3 ± 28.0	126.8 ± 26.4	119.6 ± 26.5	125.5 ± 29.5
HDL, md/dl	58.0 ± 14.1	52.1 ± 13.0	55.7 ± 12.8	50.4 ± 13.5	46.0 ± 12.4	49.1 ± 12.6
Adipokines						
Leptin, ng/ml	25.4 ± 23.5	18.1 ± 21.8	21.4 ± 27.4	24.0 ± 23.0	16.2 ± 15.6	20.4 ± 20.9
Resistin, ng/ml	6.0 ± 2.7	6.9 ± 2.8	5.5 ± 2.3	5.4 ± 2.1	6.5 ± 2.5	5.4 ± 2.4
CRP, ng/pl	4.1 ± 4.8	3.7 ± 4.3	4.3 ± 6.1	5.0 ± 6.4	3.9 ± 4.3	4.3 ± 6.4
Dietary intake						
Energy intake, kcal	2040.4 ± 599.0	1558.2 ± 440.0		2219.0 ± 572.8	1690.0 ± 468.7	
Fat, %	35.7 ± 4.9	17.5 ± 3.0		36.6 ± 5.0	31.4 ± 5.9	
Carbohydrates, %	43.9 ± 6.2	47.3 ± 7.0		42.9 ± 6.5	45.4 ± 6.8	
Protein, %	15.4 ± 3.3	17.5 ± 3.0		15.2 ± 2.7	17.8 ± 3.6	
Fibers, g/day	17.9 ± 7.4	20.5 ± 7.4		17.9 ± 6.0	21.2 ± 9.3	

Data are shown as mean±SD.

Abbreviations: ALT, alanine transaminase; AST, aspartate transaminase; GGT, gamma-glutamyl transpeptidase; HDL, high density lipoprotein; HOMA-IR homeostatic model of insulin resistance; LDL, low density lipoprotein; SAT subcutaneous adipose tissue; VAT, visceral adipose tissue
